# Comparing preoperative and postoperative dexamethasone effects on analgesia duration in shoulder surgery

**DOI:** 10.1016/j.isci.2024.109019

**Published:** 2024-01-24

**Authors:** Cheng Xu, Chengyu Wang, Yanling Hu, Fei Gu, Jie Lu, Quanhong Zhou

**Affiliations:** 1 Department of Anaesthesiology, Shanghai Sixth People’s Hospital Affiliated to Shanghai Jiao Tong University School of Medicine, 600 Yishan Road, Shanghai, China; 2 Department of Critical Care, Shanghai Sixth People’s Hospital Affiliated to Shanghai Jiao Tong University School of Medicine, 600 Yishan Road, Shanghai, China

**Keywords:** Health sciences, Medicine, Medical specialty, Surgery, Orthopedics

## Abstract

Dexamethasone is commonly used as an adjuvant to prolong peripheral nerve block analgesia, but the optimal timing is unclear. This randomized equivalence trial tested whether preoperative versus postoperative intravenous dexamethasone have equivalent analgesic effects when combined with interscalene brachial plexus block for shoulder surgery. 168 patients were randomized to receive 5 mg dexamethasone either preoperatively or postoperatively. The primary outcome was duration of analgesia, analyzed for equivalence with a 2-h margin. The mean durations were equivalent between groups (11.5 h preoperative versus 10.7 h postoperative). The confidence intervals fell within the equivalence margin. There were no other clinically significant differences in secondary outcomes like time to first analgesia, motor recovery, opioid consumption, blood glucose, or complications. In conclusion, as an adjuvant for nerve block, preoperative and postoperative intravenous dexamethasone provide equivalent analgesic duration, allowing for flexibility in clinical use. This addresses previous uncertainty about timing while demonstrating equivalent efficacy.

## Highlights

• Pre-op/post-op dexamethasone equally effective for surgical pain relief
• Analgesia lasts 11.5h pre-op, 10.8h post-op; no significant effect difference
• Timing of dexamethasone does not impact recovery or side effects post-surgery

## Graphical abstract

**Figure:**
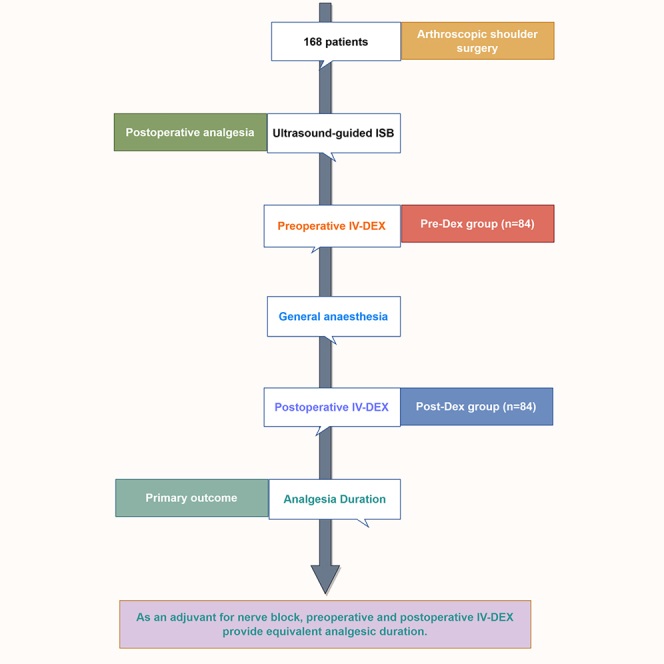

## Introduction

Arthroscopic shoulder surgery is a common orthopedic procedure that often requires regional anesthesia, such as an interscalene brachial plexus block (ISB), to provide effective pain relief during and after surgery.^1^^,^^2^ Although ISB is an effective method of pain control, the analgesic duration of single-shot ISB has been reported to be short-lived.^3^ Patients may experience postoperative rebound pain after the effect wore off, which can negatively affect their recovery and quality of life.^4^^,^^5^

Dexamethasone, a corticosteroid, is an efficacious local anesthetic adjuvant (both intravenous and perineural routes) for prolonging the duration of peripheral nerve blocks (PNBs) with minimal side effects.^6^^,^^7^ It is thought to reduce inflammation and pain by inhibiting the production of inflammatory cytokines and increasing the release of anti-inflammatory cytokines.^8^^,^^9^^,^^10^^,^^11^^,^^12^ Although controversial, the intravenous route of adjuvant dexamethasone seems more reasonable, sensible and beneficial. Contemporary literatures also call for caution in the off-label use of perineural routes due to the lack of knowledge of the local mechanism of action.^13^^,^^14^^,^^15^^,^^16^

The optimal timing of intravenous adjunct dexamethasone (IV-DEX) for PNB analgesia remains uncertain. Clinically, IV-DEX is recommended for prophylactic use to prevent postoperative nausea and vomiting (PONV) and prolong PNB duration.^6^^,^^7^^,^^13^^,^^14^^,^^17^ However, no studies have examined whether delayed IV-DEX intervention could have a similar adjuvant effect. Notably, as most rotator cuff surgeries are performed on an outpatient basis, an extended window of IV-DEX may have potential benefits for perioperative analgesia and rapid recovery. If delayed IV-DEX administration achieves a similar clinical outcome as the prophylactic strategy, it may offer more convenience in the timing of IV-DEX administration.

This trial was specially designed and powered as an equivalence trial, with a priori-defined 2-h equivalence margin, to test the hypothesis that preoperative IV-DEX is equivalent to postoperative IV-DEX on analgesic outcomes when combined with ISB using ropivacaine.

## Methods

### Trial design and participants

Between January 2021 and July 2021, this randomized, equivalency trial was undertaken at Shanghai Sixth People’s Hospital, a tertiary academic hospital connected with Shanghai Jiaotong University. This trial was approved by the Shanghai Jiaotong University Affiliated Sixth People’s Hospital Ethics Committee (No. 2020-186-(1)). This study was registered on www.clinicaltrials.gov prior to first patient enrollment (registration number: NCT04714112; https://clinicaltrials.gov/ct2/show/NCT04714112?term=NCT04714112&draw=2&rank=1; principal investigator: Quanhong Zhou; registration date: January 19, 2021). This report follows the Consolidated Standards of Reporting Trials (CONSORT) guidelines. The first patient was admitted to the hospital on January 21, 2021. All participants provided written informed consent.

For this study, adult patients, aged 18 to 70 years old, with American Society of Anesthesiologist (ASA) physical status 1 to 2, scheduled for elective unilateral arthroscopic shoulder surgery (Rotator cuff repair or/and acromioplasty) between January 2021 and July 2021, were eligible for enrollment and recruited by research assistants during preoperative anesthesia visits. Exclusion Criteria were as follows: lack of patient consent; allergy to ropivacaine; diabetic patients with poorly controlled blood sugar (>10 mmol L^−1^); BMI >35 kg/m^2^; contraindications to low dose dexamethasone (peptic ulcer disease, systemic infection, glaucoma, active varicella/herpetic infections and diabetes mellitus); contraindications to ISB, such as severe chronic obstructive pulmonary disease (COPD) with forced expiratory volume <40% predicted, coagulopathy (a pre-existing neurologic deficit in ipsilateral upper extremity and localized infection), pregnant or nursing females, chronic opioid use defined as > 30mg oral morphine or equivalent per day, and inability to understand numeric rating scale.

### Randomization and blinding

Patients providing informed consent and meeting all eligibility criteria were randomized to preoperative or postoperative groups receiving preoperative intravenous Dexamethasone (Pre-Dex group) or postoperative intravenous Dexamethasone (Post-Dex group). The computer generated a randomization sequence and sequential numbered. Sealed, opaque envelopes were prepared by an individual not otherwise involved in the study to maintain blinding and assignment concealment. Eligible participants were randomized using the numbers inside the envelopes on the day of surgery. All participants intravenously received two 5mL doses of the testing drug, one for preoperative use right after the ISB and the other in the post-anesthesia care unit (PACU). The testing drug was normal saline with or without 5mg (1mL) dexamethasone prepared by a nurse who otherwise was not involved in the study. All other personnel, including subjects, anesthesiologists who performed ISB and intraoperative management, surgeons, investigators, and statisticians who performed outcome measures, were blinded to group allocations until data analysis was completed.

### Anesthesia protocol

All subjects did not take any preemptive analgesics preoperatively. Before the study procedure, they were trained on using the patient control intravenous analgesia (PCIA) pump and instructed to use a numeric rating scale (NRS; 0 is no pain, and 10 is the worst pain imaginable).

Upon arrival in the operating room, an 18-gauge IV cannula was inserted into a peripheral vein in the unaffected arm, and an infusion of lactated Ringer’s solution was started. Three-lead ECG, heart rate, invasive blood pressure, respiratory rate, and pulse oximetry were continuously monitored. The preoperative ISB was performed by the same staff regional anesthesiologist. Typically, intravenous midazolam 1mg is used to induce sedation for ISB. After sterile skin preparation (the lateral part of the neck, including the supraclavicular fossa on the same side of the surgical site) with a solution of chlorhexidine 2% in isopropyl alcohol 70%, a sterile 50 mm 22 gauge needle was inserted in-plane until the needle tip was adjacent to the C5 and C6 roots under ultrasound guidance (MTurbo; SonoSite Inc., Bothell, WA, USA). After negative aspiration, 10 mL of 0.3% ropivacaine was injected. ISB was performed by the same experienced anesthesiologist. A 5mL testing drug (normal saline with or without 5mg dexamethasone) was administered right after the block.

General anesthesia was induced using a standardized technique of sufentanil 0.2 μg kg^−1^, propofol 1-3 mg kg^−1^, and rocuronium 0.6 mg kg^−1^. Endotracheal intubation was performed after the rocuronium was fully effective. Anesthesia was maintained with sevoflurane at a 1.4–2 vol % end-tidal concentration. All procedures were performed by one of the same three senior doctors to maximally decrease potential bias due to procedural factors: intraoperatively, intravenous sufentanil 5-10μg when blood pressure or heart rate exceeds 20% of the preoperative baseline value; bradycardia (<50 bpm) was treated with 0.25–0.5 mg atropine; standardized antiemetic prophylaxis with intravenous droperidol 1–2 mg at the end of surgery. When the patient was fully awake and resumed spontaneous breathing, the tracheal tube was removed by the anesthesiologist responsible for intraoperative anesthesia management and then transferred to the PACU. All subjects were admitted to the ward until they met the PACU discharge criteria.^18^ Before leaving the PACU, they received an intravenous 5mL testing drug (with or without 5mg dexamethasone). In order to ensure the accuracy of the tests, the interval between the two testing drugs was over 2 h.

All patients received PCIA for postoperative analgesia. PCIA protocol was programmed with 150ug sufentanil diluted to 150mL (2mL bolus, 15min lockout time interval, 1 h limit of 8mL without any baseline infusion). The patients received 50mg flurbiprofen ester intravenously every 12 h as a routine practice in the ward. All outcomes were assessed by an investigator blinded to group assignments. The investigator followed up on the evening of the surgery (6–8 h postoperatively) to re-educate subjects and/or their caregivers on the NRS score in the diary Caregivers were instructed to use PCIA for NRS>3 or on patient request. The visits were performed again in the mornings of the next two days for data collection and PCIA pump collection. Patients were discharged on postoperative day 3, per the institution’s normal routine, if no complications occurred. Telephone follow-up was conducted one week after surgery for possible complications.

### Outcomes

The primary outcome was the duration of analgesia, defined as the time (hours) from completion of ISB to the first sensation of pain at the surgical site. The secondary outcomes were: (1) time (hours) to the first request for analgesics with PCIA; (2) time of motor recovery (defined as complete recovery of motor function in the fingers, wrist on the nerve block side); (3) opioid consumption at 12h, 24h, 36h, and 48h; (4) NRS for resting pain at 12h, 24h, and 48h; (5) ISB-related complications: postoperative dyspnea, block site infection, block-related persistent paresthesia at one week after surgery; (6) other complications: PONV; (7) preoperative and postoperative fasting blood glucose levels.

### Sample size calculation

Based on our clinical experience in performing ultrasound-guided ISB (0.3% ropivacaine 10 mL) and the previous research study,^12^ we anticipated that the average duration of sensory block would be 10 h (with a standard deviation of 4 h). Furthermore, we hypothesized that the preoperative administration of intravenous dexamethasone would result in a 40% increase in the mean duration of ISB. To reliably test this hypothesis with a margin of 2 h equivalence, we calculated that a total of 138 participants (69 per group) would need to be randomly assigned (with an alpha level of 0.05 and power of 80%). To account for a potential 20% loss of follow-up, we aimed to recruit a total of 180 participants, with 90 individuals in each group. The sample size calculation was based on two commonly used equivalence analysis methods: the two one-sided tests (TOST). On the day prior to surgery, our research assistants conducted the recruitment and consent process. Finally, only patients who met all inclusion criteria were randomized on the day of surgery.

### Statistical analysis

To summarize and express patient data, appropriate central tendency and dispersion measures, as well as counts and percentages for categorical data, were utilized. For the primary outcome, which was the time to the first sensation of pain at the surgical site, the two one-sided tests (TOST) method was employed to evaluate for equivalence. Specifically, we examined whether the upper and lower bounds of the 95% confidence interval (CI) were both below the defined equivalent bounds. If this criterion was met, then the two groups were considered to be equivalent. The equivalence margin of 2 h was specified based on what we deemed to be a clinically significant difference in block time, taking into account the reported prolongation of ISB associated with dexamethasone. Adjusted factors: gender, age, BMI, ASA, Intraoperative sufentanil usage and time of surgery.

Continuous measures (opioid consumption, NRS, and blood glucose) were assessed with t-tests after the data were confirmed to be generally distributed for secondary outcomes. For the time to first analgesic request and motor recovery, the Kaplan-Meier method was used to analyze data, and the log rank test was applied to compare the two groups. Fisher’s exact test compared categorical outcomes (postoperative dyspnea, paresthesia, infection, and PONV). p < 0.05 value was considered to be statistically significant. All statistical analyses were performed using R version 3.6.3 and Python version 3.7.

## Results

### Study patients

**Figure 1:**
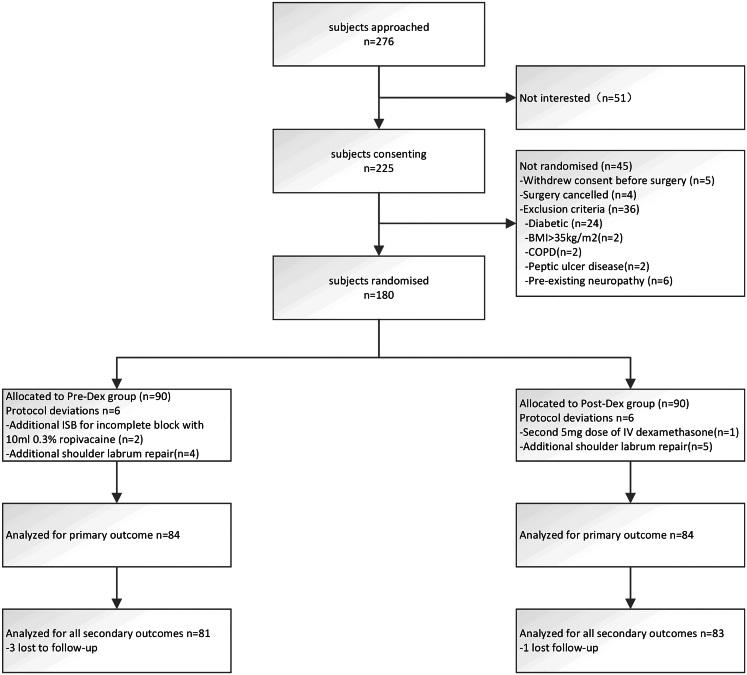
CONSORT flow diagram of patient enrollment, inclusion and exclusion process CONSORT, Consolidated Standards of Reporting Trials.

There were 264 patients admitted to the hospital for elective unilateral arthroscopic shoulder surgery (rotator cuff repair or/and acromioplasty). Among them, 51 were not interested in the study and 36 were excluded from the study because of the exclusion criteria. Of the remaining 189 patients who provided consent, five withdrew their informed consent before surgery, and four patients’ surgeries were canceled. 180 patients were randomly assigned, with 90 to the Pre-Dex Group and 90 to the Post-Dex group. The Consolidated Standards of Reporting Trials flow diagram describing the progression of patients through the study phases is shown in Figure 1.

There were twelve protocol deviations. Two subjects in the Pre-Dex Group received a second preoperative ISB because of the incomplete primary block (C5 anatomical variation). One patient in the Post-Dex Group inadvertently received a second 5mg dexamethasone after returning to the ward, and nine participants (4 in the Pre-Dex group and 5 in the Post-Dex group) underwent additional shoulder labrum repair. The primary outcome was recorded for all 168 subjects randomized. Despite multiple attempts, four participants (3 in the Pre-Dex group and 1 in the Post-Dex group) were lost to follow-up at 1-week after the surgeries, and part of the secondary outcome data for these participants was not recorded. The clinical characteristics of the 168 patients are summarized in Table 1.

**Table 1:** Clinical characteristics

Characteristics	Groups		P-value
	Pre-DEX (n = 84)	Post-DEX (n = 84)	
Age (Y) Mean ± SD	49.7 (12.7)	47.5 (10.6)	0.23
Sex: No. (%)			0.88
Male	50 (60)	49 (58)	
Female	34 (40)	35 (42)	
BMI (kg/m2)	23.9 (2.93)	23.6 (2.66)	
ASA physical status: No. (%)			0.15
1	24 (28.6)	16 (19.0)	
2	60 (71.4)	68 (81.0)	
Duration of Surgery (min)	160.4 (19.68)	164.4 (20.35)	0.19

Data are presented as mean (standard deviation) or n [%]. ASA: American Society of Anesthesiologists physical status classification; ISB: interscalene block. Fisher’s exact test for sex and ASA and the unpaired t-test for Age, BMI and duration of surgery, and other variants.

### Main results

**Figure 2:**
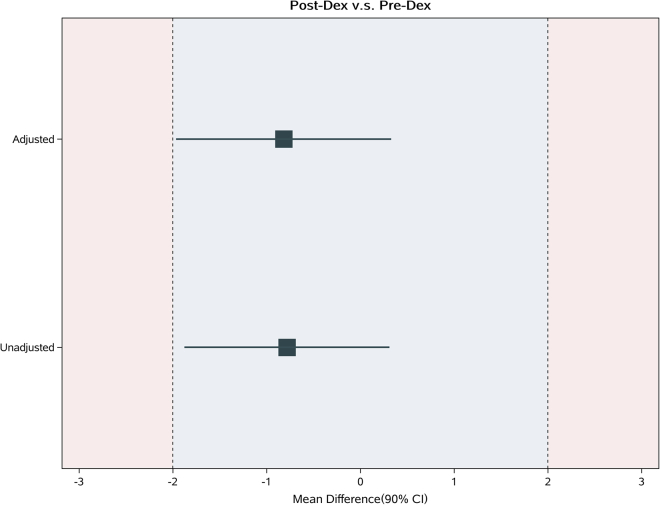
Ninety percent confidence interval of mean duration of analgesia LEL, Lower Equivalence Limit; UEL, Upper Equivalence Limit.

**Figure 3:**
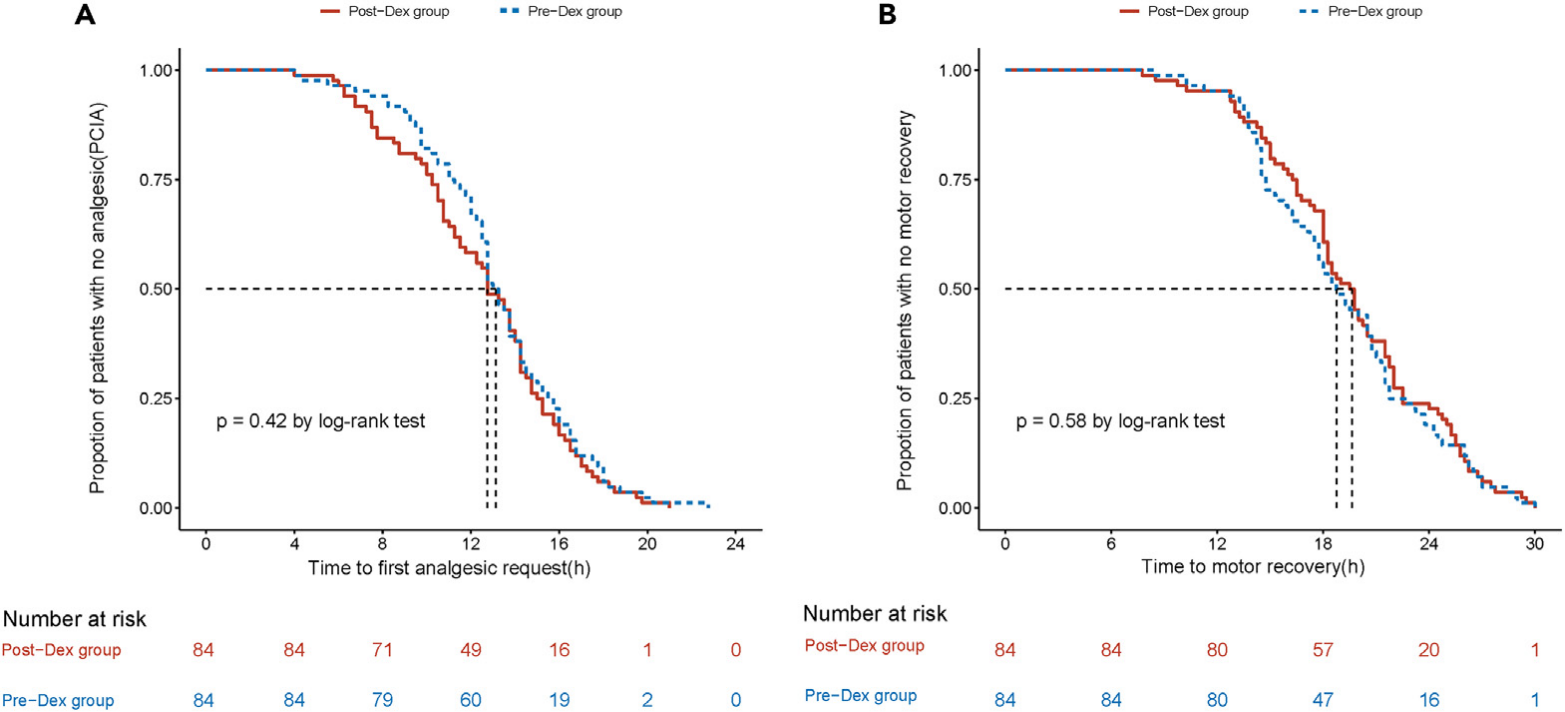
Survival curves for analgesia and motor recovery between treatment groups Kaplan-Meier estimates of the proportion of patients with (A) the first request for analgesic with PCIA in the two groups, (B) the time of motor recovery in the two groups. PCIA, Patient Control Intravenous Analgesia.

For the primary outcome, the duration of analgesia between the Pre-Dex and Post-Dex groups was equivalent to the TOST method. (Figure 2; Table 2) The observed mean (standard deviation) analgesic duration in the Pre-Dex group was 11.5 (4.12) h and 10.7 (4.45) h in the Post-Dex group, with an unadjusted mean difference of 0.78h. The upper and lower bounds of 95% CI were −1.88h and 0.31h, respectively. Adjusted mean difference (95%CI) is −0.82h (−1.97, 0.33) (Table 3). Using Kaplan-Meier survival analysis, the first request for analgesia with PCIA and the time of motor recovery (log rank test, p = 0.42 and p = 0.58, respectively) were also similar between the two groups (Figures 3A and 3B).

**Table 2:** Perioperative characteristics

	Pre-DEX	Post-DEX	P-value
**Primary outcome**			

Duration of analgesia; hrs	11.5 (4.12)	11.8 (4.45)	0.48

**Secondary outcomes**			

Total use of sufentanil	27.5 (25,30)	30.0 (20,30)	0.68
Time to first analgesic intake after ISB (hrs)	14.2 (13.5,15.2)	14.5 (14.0,15.0)	0.65
Onset time of sensory block; min	4.0 (4.0,5.0)	4.0 (3.0,5.0)	0.84
Onset time of motor block; min	11.0 (10.0,12.0)	11.0 (10.0,13.0)	0.77
Duration of motor block; hrs	18.7 (1.36)	18.7 (1.31)	0.73
Visual analogue scale for pain Max (within 24h)	5.0 (4.0,6.0)	5.0 (4.0,5.0)	0.68
Postoperative oxygen saturation	97.9 (1.27)	98.0 (1.20)	0.61
Time of extubation (min)	21.6 (4.78)	22.6 (4.46)	0.53
Time in PACU (min)	66.5 (4.5)	66.7 (5.0)	0.34
Localized infection at block site	0	0	–
Pre-operative blood glucose (mmol/L)	5.2 (1.1)	5.1 (0.9)	0.80
Postoperative blood glucose (mmol/L)	6.2 (1.2)	6.5 (1.2)	0.17
Postoperative nausea/vomiting	12 (10.5)	18 (15.1)	0.23
Persistent nerve palsy	0	0	–
Dyspnea	0	0	–

Data are presented as mean (standard deviation), median (interquartile range, IQR) or n [%]. Fisher’s exact test for postoperative nausea/vomiting, persistent nerve palsy, dyspnea and localized infection at block site and the unpaired t-test for duration of analgesia, total use of sufentanil and d time to first analgesic intake, and other variants.

**Table 3:** Mean differences of duration of Analgesia(h) between treatment groups

Analysis	Pre-Dex		Post-Dex		Mean Difference (95% CI)
	Mean	SD	Mean	SD	
Unadjusted	11.5	4.12	10.7	4.45	−0.78 (−1.88, 0.31)
Adjusted					−0.82 (−1.97, 0.33)

For the mean differences of duration of analgesia, the two one-sided tests (TOST) method was employed to evaluate for equivalence. Specifically, we examined whether the upper and lower bounds of the 95% confidence interval (CI) were both below the defined equivalent bounds. Adjusted factors: gender, age, BMI, ASA, Intraoperative sufentanil usage and time of surgery.

### Secondary outcomes

**Figure 4:**
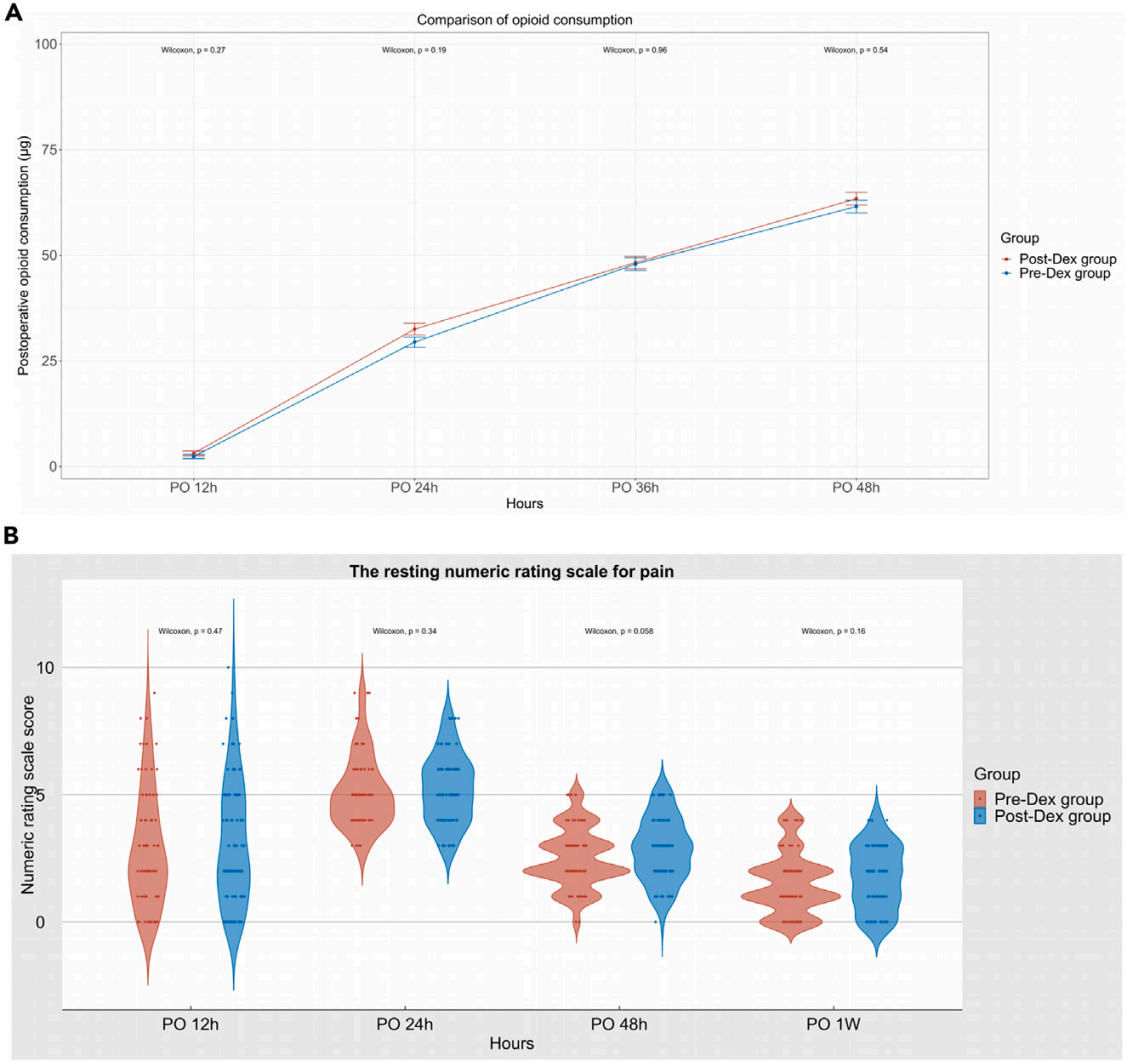
Postoperative analgesia (A) The violin diagram of postoperative opioid consumption at 12h, 24h, 36h, and 48h in the two groups. PO, Postoperative. (B) The point-line graph of numeric rating scale score for resting pain at 12h, 24h, and 48h in the two groups postoperatively. PO, Postoperative.

The mean postoperative blood glucose levels were higher than preoperative in both groups (p < 0.001). There was no statistically significant difference in opioid consumption (Figure 4A), and NRS for resting pain (Figure 4B) between the two groups at each time point. p values for the comparisons at each time point are shown in the Figure 4. No significant difference was observed between the Pre-Dex and Post-Dex groups in other secondary outcomes (Table 2).

## Discussion

This prospective, randomized, equivalent study, to the best of our knowledge, is the first trial designed to compare the effect of intravenous adjuvant dexamethasone at different times on the duration of analgesia of ropivacaine in ISB for patients undergoing arthroscopic rotator cuff repair and/or acromioplasty. We found that both preoperative and postoperative IV-DEX effectively prolonged the duration of ISB from ropivacaine. This result may provide more convenience in using dexamethasone perioperatively. Due to its late onset pharmacodynamic characteristics, dexamethasone has been recommended to be administered preoperatively to prevent postoperative nausea and vomiting.^17^ It has also been used intraoperatively to prevent postoperative inflammation and reduce postoperative tissue edema, which can be used at the end of surgery. Our study suggested that postoperative IV-DEX was equivalent to preoperative administration in prolonging the duration of ropivacaine for ISB, which could be proposed as a novel adjuvant method in postoperative analgesia.

The mechanism of action of adjuvant dexamethasone for local anesthetics appears to have a more systemic effect than a local one. While early studies indicated that perineural administration of dexamethasone prolongs the duration of PNB compared to intravenous injections,^10^^,^^11^^,^^19^^,^^20^ several more recent studies have shown that both routes produce similar analgesic effects, with intravenous administration being preferred due to potential neurotoxicity associated with perineural use.^3^^,^^21^^,^^22^ One recent study even suggested that the analgesic effects of dexamethasone are mediated systemically, as the use of a small volume of local anesthetics with perineural dexamethasone still produced systemic effects.^12^ We hypothesized that if perineural administration did not prolong blocks via the c-fibers mechanism of action, then the systemic effect of dexamethasone was likely due to its anti-inflammatory or anesthetic properties, and the timing of administration should not make a significant difference. Our results confirmed this hypothesis, as despite an almost 3-h difference in administration times, there was only a 50-min discrepancy in adjuvant effects. These findings provide additional support for the systemic mechanism of action of dexamethasone as an adjuvant for local anesthetics.

The dose of 5 mg of dexamethasone was considered based on a previous report in which the dosage was commonly used in clinical practice.^12^ And another previous study confirmed that there was no significant difference in the effects of 4mg and 8mg IV-DEX on analgesic outcomes.^20^ Although there was a dose-response association, increasing the dexamethasone dose from 8 mg to 40 mg after outpatient shoulder surgery did not significantly improve analgesia.^23^ In addition, for PONV prophylaxis, 4–8mg dexamethasone was proposed because in our institution, 5 mg/ml dexamethasone is used routinely to prevent PONV.

We used 10mL of 0.3% ropivacaine, the usual safe dose in clinical practice.^7^ Given concerns about phrenic nerve palsy due to ISB, studies have reduced the dose of local anesthetic (5 mL) with the same block success rate.^12^^,^^24^ However, the duration of analgesia is related to the total mass of local anesthetic injected.^25^ The doses of ropivacaine in other studies were much higher (20–30mL) without reporting any serious local anesthetic-related complications.^26^^,^^27^ Further, a study by Zhang et al. demonstrated no difference in the analgesic effect of 0.25%, 0.5%, and 0.75% concentrations of ropivacaine after shoulder arthroscopy.^28^ Given the concern that high concentrations of local anesthetic may lead to an increased risk of toxicity, 0.3% ropivacaine is our standard in clinical practice.

We observed significantly elevated postoperative blood glucose levels in both groups but no difference between the two groups at each time point. Although intravenous dexamethasone has been associated with postoperative impaired glucose tolerance in most studies,^29^^,^^30^ no wound healing complications were found in our study, consistent with previous studies in which dexamethasone was non-inferior to placebo concerning the incidence of surgical-site infection within 30 days after nonurgent, noncardiac surgery.^31^

### Conclusion

Preoperative and postoperative intravenous 5 mg dexamethasone provided equivalent adjuvant effects for postoperative analgesia of ropivacaine of ISB after shoulder surgeries. The results could allow for greater flexibility in the timing of the clinical use of dexamethasone.

### Limitation of the study

There are some limitations to our study. First, we assumed that dexamethasone administered within the duration of ISB of ropivacaine might produce a similar effect, so we administered dexamethasone within the block duration of ropivacaine, as reported being about 6–8 h.^4^^,^^5^ We cannot determine the optimal timing of intravenous dexamethasone without additional postoperative groups. This may need to be further verified in the future. Second, the postoperative arm bandage on the operative side limited the lateral elbow motion range, so wrist and hand movement was used as a surrogate for motor recovery. Third, we did not collect blood samples to measure serum inflammatory factor concentrations (IL-6, IL-1, TNF-α), which may have helped explore the optimal timing and the possible mechanism behind our trial results. Previous studies have confirmed the anti-inflammatory effects of intravenous dexamethasone.^32^^,^^33^ Fourth, although arthroscopic surgery often is an ambulatory procedure in most western countries, patients in our hospital prefer to stay days after shoulder arthroscopy for better care. This could be a cultural difference.

## STAR★Methods

### Key resources table

**Table:** 

REAGENT or RESOURCE	SOURCE	IDENTIFIER
**Software and algorithms**		

R version 3.6.3	R Foundation	https://www.r-project.org/
Python version 3.7	Python Foundation	https://www.python.org/

**Other**		

Ethics Committee approval (China)	N/A	Shanghai Jiaotong University Affiliated Sixth People’s Hospital Ethics Committee (No. 2020-186-(1))
Trial registration	N/A	NCT04714112

### Resource availability

#### Lead contact

Further information and requests should be directed to and will be fulfilled by the lead contact, Quanhong Zhou (zhouanny@hotmail.com).

#### Materials availability

This study did not generate new unique reagents.

#### Data and code availability

All data reported in this paper will be shared by the lead contact upon request.

This paper does not report original code.

Any additional information required to reanalyze the data reported in this paper is available from the lead contact upon request.

### Experimental model and study participant details

The study population comprised patients aged 18–70 with American Society of Anesthesiologists (ASA) physical status 1–2 for elective unilateral arthroscopic shoulder surgery (Rotator cuff repair or/and acromioplasty) between January and July 2021. The subjects were all Chinese patients. Research assistants recruited patients during preoperative anaesthesia visits. The exclusion criteria were: Contraindications to low-dose dexamethasone include lack of patient consent, allergy to ropivacaine, poorly controlled blood sugar (>10mmol L-1), BMI > 35 kg/m^2^, peptic ulcer disease, systemic infection, glaucoma, active varicella/herpetic infections, diabetes mellitus, severe COPD with predicted forced expiratory volume < 40%, and coagulopathy. All participants signed a written informed consent.

This was a randomised, equivalency trial, and was undertaken at Shanghai Sixth People's Hospital, a tertiary academic hospital connected with Shanghai Jiaotong University in China. The study protocol was approved by the Shanghai Jiaotong University Affiliated Sixth People's Hospital Ethics Committee (No. 2020-186-(1)). The protocol was amended once for minor changes (subject protection strategies have been added where appropriate). The final version of the protocol is available on demand.

Key data and resources table are available upon demand to the lead contact person.

### Method details

Patients who gave informed consent and completed all eligibility criteria were randomised to receive intravenous Dexamethasone preoperatively or postoperatively. Computer-generated randomization and sequential numbering. A non-study participant sealed, opaque envelopes to ensure blinding and assignment concealment. On operation day, eligible individuals were randomised using envelope numbers. All subjects got two 5ml intravenous doses of the testing medication, one after the ISB and one in the PACU. A non-study nurse produced normal saline with or without 5mg (1ml) dexamethasone. Group allocations were hidden from subjects, ISB and intraoperative management anesthesiologists, surgeons, investigators, and outcome measure statisticians until data analysis.

The staff regional anesthesiologist did the preoperative ISB. ISB sedation usually involves intravenous midazolam 1mg. After negative aspiration, 10 ml 0.3% ropivacaine was administered. The same skilled anesthesiologist performed ISB. The block was followed by a 5ml testing drug (normal saline or 5mg dexamethasone). A standardised approach of sufentanil 0.2 μg kg-1, propofol 1-3mg kg-1, and rocuronium 0.6 mg kg-1 was used to achieve general anaesthesia. Endotracheal intubation followed rocuronium's full action. Sevoflurane at 1.4-2 vol% end-tidal sustained anaesthesia. The intraoperative anesthesiologist withdrew the tracheal tube and transferred it to the PACU once the patient was awake and breathing spontaneously. The ward admitted all subjects until they met PACU discharge criteria. They got a 5ml intravenous testing medication with or without 5mg dexamethasone before leaving the PACU.

Every patient received PCIA for postoperative analgesia. The ward routinely gave patients 50mg flurbiprofen ester intravenously every 12 hours. An investigator blinded to group allocations analysed all outcomes. The investigator reminded participants and carers of the NRS score in the diary on the evening of operation (6-8 hours postoperatively). Carers were told to use PCIA for NRS-3 or patient request. The following two mornings, data and PCIA pump collection were repeated. The institution's typical discharge was on postoperative day 3 if no difficulties arose. One week following surgery, problems were checked by phone.

### Quantification and statistical analysis

Analgesia duration, measured in hours from ISB completion to surgical site pain, was the primary outcome. The secondary outcomes were: (a) time (hours) to the first request for analgesics with PCIA; (b) motor recovery (complete recovery of motor function in the fingers, wrist on the nerve block side); (c) opioid consumption at 12h, 24h, 36h, and 48h; and (d) NRS for resting pain at 12h, 24h, and 48h.

Based on our clinical experience with ultrasound-guided ISB (0.3% ropivacaine 10 ml) and the prior research study12, we expected sensory block to last 10 hours (with a 4-hour standard variation). We also hypothesised that preoperative intravenous dexamethasone would increase ISB duration by 40%. With an alpha level of 0.05 and power of 80%, we determined that 138 participants (69 per group) would need to be randomly assigned to test this hypothesis with a two-hour equivalency margin. To account for a 20% loss of follow-up, we recruited 180 participants, 90 per group. The sample size was calculated using two common equivalency analysis methods: two one-sided tests.

Results are presented as count (%), mean ± standard deviation or median (25-75th percentile) depending on data type and distribution. For the primary outcome, which was the time to the first sensation of pain at the surgical site, the two one-sided tests (TOST) method was employed to evaluate for equivalence. Results are presented in tables and graphs. All statistical analyses were performed using R version 3.6.3 and Python version 3.7.

### Additional resources

This study was registered on www.clinicaltrials.gov prior to first patient enrollment (registration number: NCT04714112; principal investigator: Quanhong Zhou; registration date: January 19, 2021).
